# Intracranial Hemorrhage During Pregnancy: An Interdisciplinary Literature Review and a Rare Case Report of Early-Onset Eclampsia with Intracranial Hemorrhage and HELLP Syndrome

**DOI:** 10.3390/jcm14041361

**Published:** 2025-02-18

**Authors:** Natalia Katarzyna Mazur, Justyna Małgorzata Fercho, Maria Kałas, Karolina Szaruta-Raflesz, Magdalena Emilia Grzybowska, Mariusz Siemiński, Dariusz Grzegorz Wydra

**Affiliations:** 1 Department of Gynecology, Obstetrics and Neonatology, Medical University of Gdansk, 80-210 Gdansk, Poland; magdalena.grzybowska@gumed.edu.pl (M.E.G.); dariusz.wydra@gumed.edu.pl (D.G.W.); 2 Clinic of Obstetrics and Gynecology, Gynecological Oncology and Endocrine Gynecology, University Clinical Centre, 80-952 Gdansk, Poland; 3 First Doctoral School, Medical University of Gdansk, 80-210 Gdansk, Poland; 4 Department of Neurosurgery, 10th Military Hospital, 85-681 Bydgoszcz, Poland; jfercho@uck.gda.pl; 5 Clinic of Emergency Medicine, University Clinical Centre, 80-952 Gdansk, Poland; maria.kalas@gumed.edu.pl (M.K.); karolina.szaruta-raflesz@gumed.edu.pl (K.S.-R.); mariusz.sieminski@gumed.edu.pl (M.S.); 6 Department of Emergency Medicine, Medical University of Gdansk, 80-210 Gdansk, Poland

**Keywords:** intracranial hemorrhage, eclampsia, preeclampsia, HELLP syndrome, tonic–clonic seizures, emergency medicine, neurosurgery, obstetrics, pregnancy

## Abstract

Intracranial hemorrhage is a rare yet potentially devastating event during pregnancy with a significant risk of maternal and fetal mortality and morbidity. The risk of intracranial hemorrhage increases during the third trimester of pregnancy and is greatest during labor and the postpartum period. Interdisciplinary diagnosis and treatment of the pregnant population often begins in the emergency department setting and is key to increasing patient survival rates through immediate and adequate treatment, including emergency medicine, neurosurgical and obstetrical procedures. A unique case report with a diagnostic pathway for intracranial hemorrhage due to eclampsia in a primipara at 24 weeks of gestation is presented, illustrating potential diagnostic dilemmas as the patient rapidly progresses into hemolysis, elevated liver enzymes and low platelets syndrome. A literature review was conducted to uncover the etiology of intracranial hemorrhage during pregnancy, as well as its diagnostic challenges and treatment. Pregnancy should not be viewed as a barrier to performing angiography or endovascular treatment for vascular causes of intracranial hemorrhage. Patient transport to a tertiary reference center and the interdisciplinary cooperation of specialists are key to achieving correct and rapid treatment. Continuous prevention of preeclampsia and patient education are necessary to decrease the incidence of eclampsia and its complications. Key message: Intracranial hemorrhage and eclampsia in pregnant patients are rare yet may result in high rates of maternal and fetal morbidity and mortality. The diagnostic process is difficult and requires interdisciplinary cooperation to start the correct treatment immediately.

## 1. Introduction

Intracranial hemorrhage (ICH) is a rare yet potentially devastating event in pregnancy. The maternal and fetal mortality and morbidity resulting from this event are high, and the risk of hemorrhage increases in the last trimester of pregnancy and is highest during labor and the postpartum [1]. Advanced maternal age, hypertension disorders, coagulopathy, preeclampsia or eclampsia and tobacco use are independently associated with ICH during the pregnancy and postpartum period [1].

Aneurysmal subarachnoid hemorrhage (aSAH) is reported as one of the main causes of ICH in pregnancy [2]. According to the World Federation of Neurological Surgeons (WFNS)’s grading system, poor-grade aneurysmal subarachnoid hemorrhage (aSAH) (Grades IV and V) accounts for 20–30% of all aSAH, specifically in a state of coma or depressed consciousness.

Patients who retain a preserved level of consciousness typically report a sudden onset of severe headache, often described as the worst headache of their life, accompanied by vomiting. This symptomatology may mimic that of eclampsia or preeclampsia, particularly in women of childbearing age, which underscores the importance of accurate diagnosis in emergency settings.

The authors aim to describe and analyze intracranial hemorrhage in the pregnant population from etiopathology to differential diagnosis and treatment. Upon analysis, a rare case report will be dissected to underline specific challenges facing not just the obstetrician and gynecologist (OB/GYN) in the hospital setting but also other medical professionals in the prehospital setting, emergency department and neurosurgery, who are often first treating the patient.

The rarity of ICH during pregnancy, its acute onset and its progressive symptoms are important enough that they must be described not just to OB/GYN and midwife professionals, but to primary care providers who may have little experience with pregnant patients. As eclampsia and preeclampsia (PE) are often the cause of ICH, a whole spectrum of pregnancy-specific disorders may arise shortly following or concomitantly, including hemolysis, elevated liver enzymes and low platelets (HELLP) syndrome, and disseminated intravascular coagulation (DIC).

## 2. Material and Methods

On 15 November 2024, the MEDLINE (PubMed) database was searched manually by two authors (J.M.F. and N.K.M.). The eligibility criteria for article analysis were ICH in the pregnant population and a date of publication in the last 24 years (2000–2024). Only full-text articles in the English language were considered for further analyses. The search formula was “ICH” AND “pregnancy”. The database search provided 522 unique results, and authors rejected 513 articles after title and abstract screening due to incomplete data or because the English version of the article was not available. Finally, 11 articles were included in the narrative literature review, comprising a population-based cohort estimate study, systematic reviews, questionnaire-based population studies and a series of case reports.

A case report is described in detail with the differential diagnosis, laboratory and imaging results in the discussion. The patient’s symptoms and consequent treatment illustrate real-time decision-making by an interdisciplinary team of medical professionals, first at a secondary care center and then at several departments in a tertiary hospital.

## 3. Results

### 3.1. Hemodynamic Changes in Pregnancy and Labor

Pregnancy is a complex physiological condition that initiates profound adaptations in the cardiovascular system to support the growing fetus. During early pregnancy, there is a notable increase in blood volume that is crucial for fetal development and placental circulation. Blood volume increases by approximately 50% and plateaus around 32 weeks of gestation [3]. This expansion is attributed primarily to increased plasma volume, which supports the metabolic demands of the mother and fetus. However, it is important to note that, while blood volume increases, plasma volume may experience a relative decrease later in pregnancy due to the hemodilution effect. Cardiac output significantly increases, rising by 30–50% during the first 24 weeks of pregnancy [3]. This surge is facilitated by a decrease in systemic vascular tone, resulting in lower blood pressure. The reduction in vascular resistance allows for enhanced blood flow to vital organs and the placenta, ensuring adequate oxygen and nutrient delivery. As the body transitions into the first stage of labor, cardiac output further increases by approximately 50%. Mean arterial pressure can rise by up to 20% during uterine contractions, reflecting the heightened cardiovascular demands during this critical time [3]. These changes are essential for managing the increased workload and maintaining uteroplacental perfusion. Remarkably, within 24 h after delivery, most hemodynamic variables return to pre-labor baseline levels.

### 3.2. ICH in Pregnancy—Etiology

The etiology of intracranial hemorrhage in pregnancy is multifactorial, with vascular malformations like arteriovenous malformations (AVM) and aneurysms being the most prevalent causes (41%). Other significant contributors include preeclampsia, moyamoya disease, cavernous angioma, cerebral venous sinus thrombosis (CVST) and tumors. Less common causes and conditions that may predispose patients to ICH include coagulopathies as well as infections. A portion of cases remain unexplained [4,5]. Awareness of these conditions is essential for healthcare providers to ensure prompt diagnosis and management, ultimately improving maternal and fetal outcomes.

### 3.3. Arteriovenous Malformations Bleeding in Pregnancies

The prevalence of AVMs is estimated to be between 15 and 18 per 100,000 individuals [6]. The maternal mortality rate following hemorrhage from an AVM is reported to be 28% [7]. The estimated risk of hemorrhage from previously unruptured AVMs in pregnant women is around 3.5%. In comparison, the risk of hemorrhage in non-pregnant women of childbearing age is slightly lower, at 3.1% [7]. This indicates that pregnancy does not significantly increase the risk of hemorrhage from unruptured AVMs. For women who have already experienced a hemorrhage from an AVM, the risk of further hemorrhage is reported to be 27% [7]. This rate is notably higher than the rebleeding rate observed in non-pregnant patients, which is 7.6% [8]. These findings highlight the complexities involved in managing AVMs during pregnancy, and suggest that, while the overall risk of hemorrhage may not be significantly elevated in pregnant women with unruptured AVMs, those who have experienced a hemorrhage face a higher risk of rebleeding.

### 3.4. Preeclampsia, Eclampsia and Cerebral Autoregulation

Preeclampsia is a complex pregnancy-related disorder characterized by the onset of hypertension and proteinuria after 20 weeks of gestation. Recent studies utilizing animal models have elucidated critical pathophysiological mechanisms underlying this condition, particularly focusing on impaired cerebral autoregulation and alterations in blood–brain barrier (BBB) integrity [9]. Normal cerebral blood flow (CBF) as it relates to cerebral perfusion pressure (CPP) remains fairly stable, within the range of 60 to 150 mmHg of CPP. When the pressure goes beyond these thresholds, autoregulation is compromised, causing CBF to vary directly with pressure. In cases of chronic hypertension, the autoregulatory curve is adjusted to higher pressures. During normal pregnancy, there may be a shift in the autoregulatory curve, which is believed to contribute to the occurrence of eclampsia in some women at standard blood pressure levels. The loss of autoregulation, where CBF adjusts linearly with pressure, is thought to take place during eclampsia [10].

### 3.5. ICH Management During Pregnancy

The main objective of managing ICH is to reduce the likelihood of rebleeding [2]. It is especially important for pregnant patients, given the implications for both maternal and fetal health. Diagnosis and treatment strategies must be meticulously customized to balance the need for immediate action with possible risks to the fetus. Employing techniques such as magnetic resonance angiography, computer tomography (CT) angiography or digital subtraction angiography to evaluate vascular conditions and identify any underlying causes of ICH is crucial. For unruptured aneurysms, MR without contrast, particularly time-of-flight MR angiography, is a viable option for characterizing the lesion while limiting fetal exposure to contrast and radiation. Pregnancy should not be seen as a barrier to performing angiography and endovascular procedures when investigating a vascular source of ICH [2]. It is well-established that radiation exposure during pregnancy can pose risks to the developing fetus. While a single abdominal CT scan may not significantly increase the risk of miscarriage or congenital malformations, it can potentially double the risk of childhood cancer [11]. Therefore, minimizing radiation exposure is essential, but in critical situations, the necessity of the procedure may justify the risk. The use of contrast agents in imaging procedures also raises concerns about fetal exposure. Some contrast agents can cross the placenta and may pose risks to the fetus. It is important to use the lowest effective dose and to consider alternatives when possible. In addition to the above-mentioned, here we present some other effective strategies to minimize fetal exposure: avoiding multiphase examinations, limiting coverage to the region of interest, increasing pitch, widening collimation, decreasing tube voltage and applying abdominal shielding [12]. It is crucial to recognize the potential risks to the fetus, including exposure to radiation, contrast agents, and maternal blood loss, which may jeopardize the health of both the mother and child. In cases of ruptured aneurysms, prompt intervention is necessary, utilizing the most effective treatment methods regardless of the patient’s pregnancy status. A ruptured aneurysm should be treated urgently based on the best available option for the patient—endovascular coiling or surgical clipping—regardless of her pregnancy status [2,4]. Managing hypertension is also vital; maintaining blood pressure below 140/90 mmHg can help mitigate the risk of rebleeding [13]. An urgent collaborative approach is essential, involving obstetricians, neurologists, and interventional radiologists, to evaluate maternal safety and outcomes. Treatment decisions should factor in the well-being of both the mother and fetus, particularly if the pregnancy has reached a viable gestational age, as neonatal outcomes are generally more promising [13]. If the pregnancy is at a non-viable gestational age, treatment should proceed as it would in non-pregnant patients to ensure maternal safety and health outcomes. It may also be advisable to expedite delivery through cesarean section (CS) to prevent Valsalva maneuvers and transient spikes in blood pressure that could lead to additional hemorrhaging [13].

In instances of AVM rupture occurring late in pregnancy, the immediate priority should be delivery. After the birth, conventional methods can be employed to treat the AVM. Vaginal delivery is possible following AVM resection, but caution is necessary, as fragile blood vessels at the resection site may increase the risk of hemorrhage in the weeks immediately following surgery [2,7].

### 3.6. Case Report

A patient aged 28 in her first uncomplicated to date pregnancy of 23 weeks and 1 day, without any prior significant medical history, was admitted to a second care level emergency department due to upper abdominal pain lasting a few hours. Her last appointment in the obstetric outpatient clinic was a month earlier. During pregnancy, the patient had a first trimester ultrasound and a cervical smear with normal findings. Her blood pressure was noted monthly during pregnancy and remained within a normal range. She did not perform any other non-invasive or invasive prenatal tests. In addition to upper abdominal pain, the patient complained about headaches, loose stools and vomiting. Upon admission, the patient’s vital signs were normal, and she consulted with an obstetrician who performed a uterine and fetal ultrasound with normal findings. Based on the reported symptoms, biliary colic or gastroenteritis were suspected; an abdominal ultrasound was performed by a radiologist and the patient consulted with a surgeon, who found no indications for surgical intervention. The patient was observed in the emergency department awaiting blood and urine test results (Table 1).

The after about 8 h of observation, the patient had two generalized tonic–clonic seizures which ceased after intravenous diazepam administration. An angio-venous brain CT was performed; a vascular malformation in the central nervous system (CNS) without evident signs of fresh hemorrhage was suspected. The patient was neurologically consulted, and it was decided that she would be transferred to a tertiary reference center. In the course of the initial hospitalization in the secondary care emergency department, pharmacotherapy consisted of diazepam, levetiracetam, kalium chloride, magnesium sulfate in bolus intravenous dose of 4 g, crystalloids and morphine.

The senior emergency physician in the tertiary center was notified about the transfer of a pregnant patient with newly onset hypertension, following a first in the lifetime tonic–clonic seizure with suspected ICH due to a vascular malformation in the CNS. Upon receiving this notification, the on-call obstetrician was informed. Methyldopa was secured and an MR of the brain was scheduled. The patient was admitted to the tertiary emergency department two hours later in a moderately poor general condition, conscious but periodically confused and appearing disoriented. Among the deviations noted in the physical examination, hypertension drew attention since the initial blood pressure was at 180/120 mmHg. The patient was transferred to the intensive care area. Antihypertensive treatment was initiated: the patient was given 10 mg of nitrendipine orally. Due to a lack of effect, 250 mg of methyldopa was added, resulting in a decrease in blood pressure to 158/108 mmHg. The patient was consulted by a neurologist and a neurosurgeon. As a conclusion, it was recommended to extend diagnostics to include MR. The neurological assessment revealed that the patient had been reporting pulsating, symmetrical, frontal headaches for a week. She denied experiencing phono- and photophobia. The intensity of headaches gradually increased. In the past, she had also suffered from headaches with migraine characteristics. Simultaneously with the implementation of the antihypertensive treatment, laboratory tests were performed in which, compared to previous measurements, the deviations described in Table 2 were noted.

Due to the abnormal results of laboratory tests, a suspicion of developing HELLP syndrome was raised. The on-duty obstetrician was informed, and a bedside consultation took place, with ultrasonography revealing a single fetus in stable condition, a fetal heart rate of 143 beats per minute, normal amniotic fluid index and normal placenta that was on the anterior uterine wall. An immediate transfer was planned to the obstetrics department after a brain MR. Simultaneously, the on-call radiologist performed a bedside ultrasound to assess the liver, which was normal and without liver hematoma. The patient’s condition was continuously monitored, with blood pressure maintained at around 120/80 mmHg. Due to fluctuating consciousness, a repeat neurological assessment was conducted, recommending maintaining anti-epileptic treatment with 250 mg of oral levetiracetam every 12 h. Further laboratory tests, a routine electroencephalogram (EEG) and a hematological consultation were conducted for the differential diagnosis of hemolytic–uremic syndrome (HUS) and thrombotic thrombocytopenic purpura (TTP).

**Figure 1:**
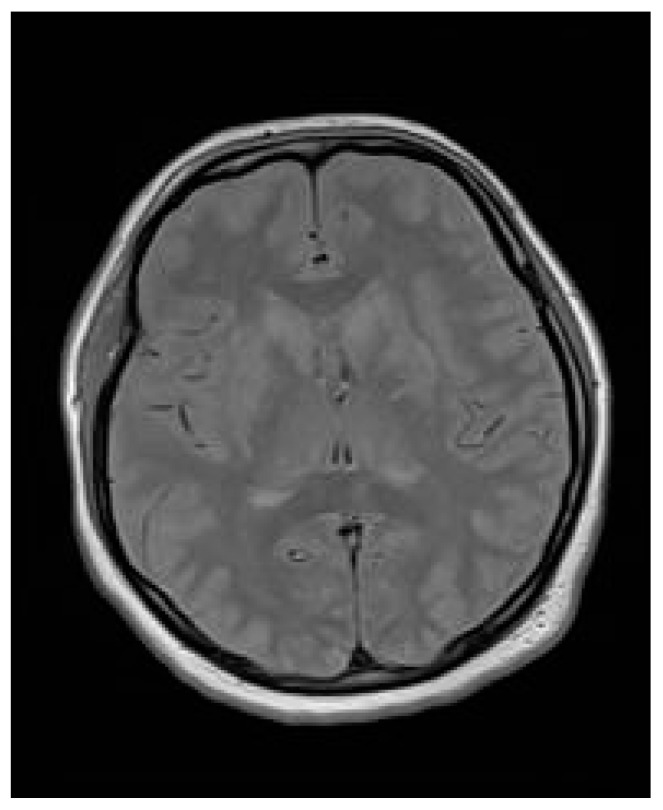
Brain MR performed at the tertiary emergency department with signs of SAH on the vault at the right parietal and left frontal lobes.

MR of the brain reveled “symmetrical, bilateral vasogenic edema of the subcortical white matter, in both cerebral hemispheres and all lobes—severe changes, causing partial tightening of the cerebral sulci with signs of subarachnoid hemorrhage (SAH) on the vault at the right parietal and left frontal lobes. The ventricular system is positioned medially, symmetrical, not dilated. No signs of compression in the posterior cranial fossa were demonstrated. Conclusion: the morphology of severe changes in the cerebral white matter primarily supports the diagnosis of posterior reversible encephalopathy syndrome (PRES), probably secondary to preeclampsia/eclampsia” (Figure 1).

**Figure 2:**
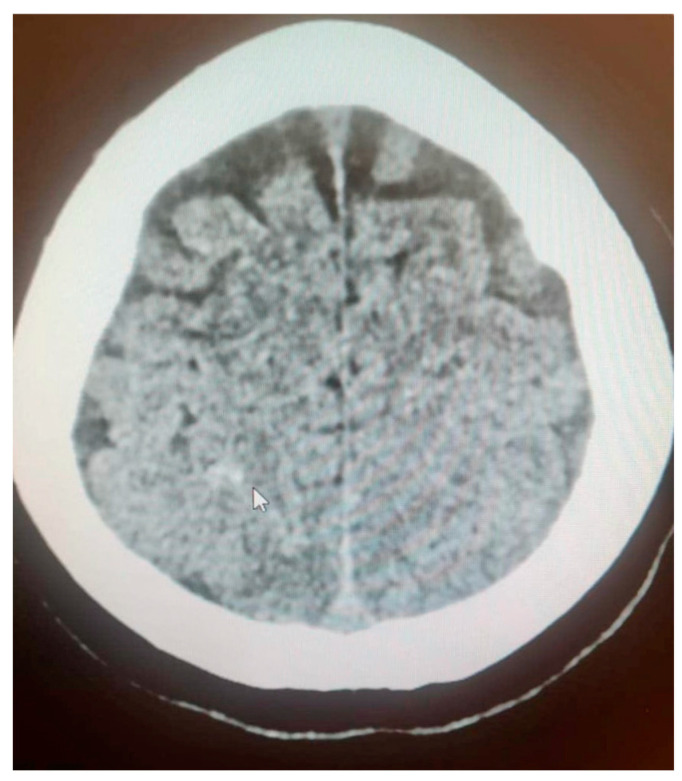
Brain CT confirmed cerebral edema, areas of hypodensity and SAH (white arrow).

**Figure 3:**
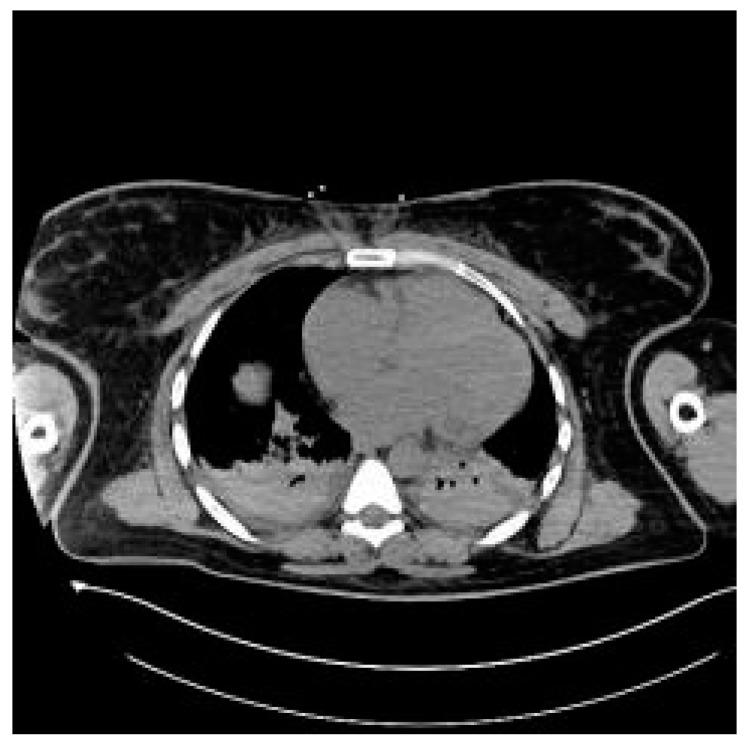
Chest CT with lung consolidations bilaterally.

The patient was transferred to the postoperative intensive care unit (ICU) in a serious general condition. A control CT of the brain confirmed cerebral edema, areas of hypodensity and SAH (Figure 2). An angio-chest CT revealed a small amount of fluid in both pleural cavities, up to 12 mm. Areas of parenchymal consolidation were visible bilaterally in the dorsal parts of the lungs, partially with an air bronchogram, which may correspond to atelectatic inflammatory changes. No signs of pulmonary embolism were found. An echocardiogram was performed, which showed good global left ventricle contractility, an estimated eviction fraction of 50%, and no signs of cardiopulmonary overload; as well, it found significant symmetrical hypertrophy of the cardia (14 mm) and traces of aortic insufficiency. An abdominal CT revealed an enlarged liver and a small amount of free fluid in the pelvis (Figure 3). Following the scans, the patient was transferred to an obstetrics operation theater, where an immediate cesarean section was performed due to high risk of maternal mortality, delivering an extremely immature, low-weight fetus. Regardless of the highest level of neontological care, fetal resuscitation procedures were abandoned, palliative care was provided and fetal death was confirmed within 2 h.

**Figure 4:**
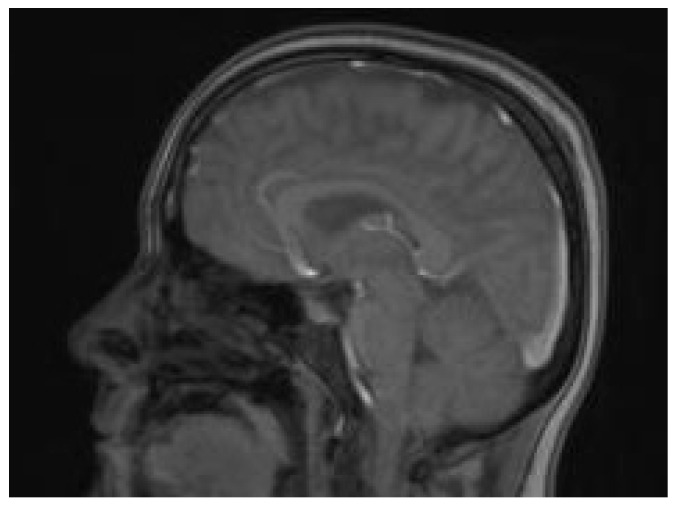
Brain MR performed after 7 days of treatment at the intensive care unit with remission in changes in the course of PRES and a reduction in cerebral edema.

Due to the serious general condition of the patient, she was transferred to the department of anesthesiology and intensive care for the treatment of acute respiratory failure and complications of hypertensive encephalopathy. A control brain MR at post-cesarean day 3 and day 7 (Figure 4) described a remission in changes in the course of PRES—a condition in which parts of the brain are affected by swelling—and a reduction in cerebral edema, which ultimately allowed for discontinuation of analgesia, sedation and extubation of the trachea on post-cesarean day 7. During hospitalization in the ICU, hematological diagnostics were performed, and the presence of circulating anticoagulants was excluded.

The patient presented persistent hypertension, initially requiring the use of a urapidil infusion, followed by methyldopa. Neurological diagnostics were expanded to include EEG, which recorded the bioelectric activity of the brain with minor changes. During ICU treatment, the patient experienced visual hallucinations, and a psychiatric consultation revealed somatogenic neurasthenia and secondary delirium disorders diagnoses with effective olanzapine treatment.

The patient was provided with continuous psychological support with satisfactory results. On day 11 of hospitalization, the patient was transferred to the obstetrics department where hypotensive, psychiatric, psychological and physiotherapeutic treatment continued with help from specialist professionals. In the course of hospitalization, laboratory results normalized, leading to her being discharged home on the 20th day in good general condition (Table 3).

**Figure 5:**
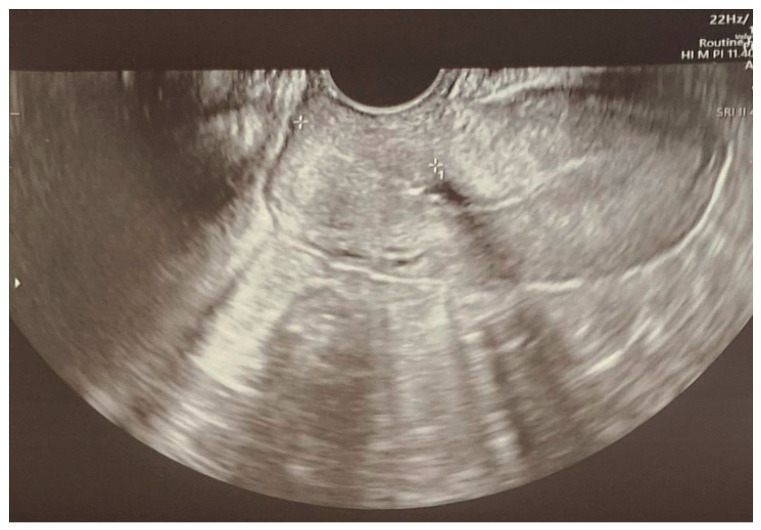
Transvaginal ultrasound revealed correct uterus healing 3 months after the cesarean section.

The discharge recommendations included outpatient OB/GYN, hypertension, neurological and psychiatric appointments and further psychological care. The pharmacotherapy prescribed included nitrendipine, beta-blockers, levetiracetam, sertraline and estazolam at night in the case of insomnia. The patient remained in the outpatient care of the tertiary hospital, and at a gynecological follow-up visit performed 3 months after the cesarean section, a transvaginal ultrasound revealed correct healing of the uterus (Figure 5). The patient sustained no long-term mental or neurological impairments, yet she remains under yearly observation. Due to normalization in her blood pressure, hypotensive treatment was stopped, and the patient was scheduled for yearly echocardiography and ophthalmological scans.

### 3.7. Case Report—Differential Diagnosis

Differential diagnosis should be actively considered and discussed at every step of patient care. Initially, the patient presented with upper abdominal pain, headaches, loose stools and vomiting while maintaining normal vital signs. Preeclampsia and eclampsia are rare causes of initially reported symptoms. Excluding gastroenteritis or appendicitis, which could have been indicated by high inflammation markers and abdominal ultrasonography, was prioritized, leading to normal findings. Another cause of initial symptoms could have been pancreatitis, which was excluded by negative lipase and amylase levels and no signs of pancreas inflammation in the abdominal ultrasound. Similarly, biliary colic was excluded based on correct bilirubin levels, normal initial liver function tests and an uneventful abdominal ultrasound.

At the same time, laboratory tests leading to HELLP syndrome diagnosis could have been performed immediately, including a complete blood count, liver function tests and lactate dehydrogenase levels. At the moment of the tonic–clonic seizures’ occurrence, magnesium sulfate should have been administered not only in a 4 g bolus, but additionally via continuous intravenous administration. The patient’s vital signs could have been taken at shorter intervals of every 30 min, which could have led to uncovering the rise in blood pressure earlier, yet we cannot be certain if it would have changed the patient’s outcome.

Upon reflection on the diagnostic process, it is key to underline that eclampsia may not be preceded by preeclampsia and that hypertension may not be present. In the case reported, screening for preeclampsia risk factors during the first trimester scan could have revealed the patient’s increased risk of early onset preeclampsia, which would have led to possibly preventative pharmacotherapy with 150 mg of acetylsalicylic acid. This finding would then increase clinicians’ vigilance and point to the right diagnosis of eclampsia when the patient presented with seizures. It should be remembered that the suspicion of ICH complicated further clinical decisions. Unfortunately, no interventions would have improved the chances of fetal survival due to the significant immaturity at 23 weeks and 1 day of gestation.

## 4. Discussion

Protocol for treating women with suspected preeclampsia in the emergency department should be aimed at confirming or excluding the risk of severe complications, such as the development of eclampsia, HELLP syndrome, pulmonary edema, cerebral edema, SAH and PRES [14]. Numerous diseases, some of which are highly specific to the pregnant population, have a similar course to preeclampsia and may coexist. It is important to actively examine patients for symptoms of infection, trauma, gastroenterological disorders and neurological disorders, as even slight alterations may lead to successful differential diagnosis [15].

Among laboratory tests, attention should be drawn to glucose levels, complete blood counts, electrolytes, renal function panels, liver function tests, fibrinogen levels, inflammation parameters and urine tests, especially with protein ratings [16]. Laboratory testing is important, but it should not delay the diagnosis and management of eclampsia. Upon patient presentation with cardiopulmonary symptoms, echocardiography and ultrasound lung assessments should be performed. In the case of neurological symptoms, it may be necessary to perform a head CT scan and consult a neurologist. It is crucial to perform a gynecological and obstetrical consultation with a fetal assessment [16,17].

The treatment taken in the emergency department setting should be immediate and appropriate to the patient’s condition and examination results. In the case of generalized seizures, the attack should be stopped as soon as possible, and administration of preventative pharmacotherapy is important.

The first-line drug for treatment is magnesium sulfate, which stops seizures and decreases the risk of their return: intravenous administration of 4–6 g of a magnesium sulfate infusion over 15 min, followed by maintenance infusions of 1–3 g/h [17]. If intravenous access is not secured, intramuscular injections of 10 g of magnesium sulfate are necessary. There are not many contraindications for magnesium apart from severe hypocalcemia, myasthenia gravis, complete heart block and myocarditis. The second-line treatment is intravenous benzodiazepines.

Magnesium sulfate treatment is considered safe, yet the serum magnesium concentration should be monitored. Loss of deep tendon reflexes can occur at blood concentrations > 3.5 mmol/L, and this is the first physical exam finding indicating magnesium toxicity. Respiratory arrest can occur when the magnesium blood concentration reaches >5–10 mmol/L, and cardiac arrest can occur at a concentration > 12.5 mmol/L. In the case of toxicity, infusion must be stopped and calcium chloride, fluids and diuretics administered immediately. If severe renal failure occurs, hemodialysis may be necessary [18]. Treatment of hypertension should be conducted with special attention. The blood pressure should not be lowered by more than 20% at a time, and the targets are a systolic blood pressure (SBP) of 130–150 mmHg and diastolic blood pressure (DBP) of 80–100 mmHg. For severe hypertension, the treatments of choice are methyldopa, labetalol, nicardipine, hydralazine and nifedipine [18].

The choice of medicine will depend on the state of the patient (i.e., whether medication can be administered orally or parenterally) and on its availability in the emergency department. It should be remembered that the above pharmacological treatment is symptomatic only, and the only causal treatment is the ending of the pregnancy, so immediate consultation with OB/GYN is crucial to decide upon the delivery method, which is most cases, will be cesarean section [17,18]. Depending on the results of additional examinations, cardiology, neurology or neurosurgical consultations may be necessary, which makes patient transfer to a tertiary hospital recommended. The prehospital setting of eclampsia has not been researched in detail to date, yet upon diagnosis of tonic–clonic seizures in the pregnant patient, the pharmacological algorithms described above should be administered, along with transfer to a hospital and, if possible, a tertiary center [19].

Finally, ongoing prevention and screening for patients with a high risk of developing preeclampsia should be practiced globally, as inexpensive and simple treatment with oral acetylsalicylic acid at a dosage of 150 milligrams administered before the 16th week of gestation may be life-saving for both the mother and fetus [20]. Patients with a history of hypertension disorders, preeclampsia or eclampsia should be regularly monitored by maternal–fetal medicine professionals, with regular screenings for symptoms and ongoing patient education of alarming signs [21,22]. It is key to underline that hypertensive disorders of pregnancy account for up to 9.4% cases of stillbirths, and a proportion of these are preventable if they receive correct diagnosis and treatment [23].

Upon recognizing one or more alarming symptoms, such as headaches, vision disturbances, high blood pressure or upper abdominal and upper right quadrant pain, the patient should be examined to exclude the potentially life-threatening, multi-organ conditions of preeclampsia, eclampsia, ICH and HELLP syndrome, which often coexist.

Interestingly, few cases of ICH during pregnancy present with similarities to the case reported, yet Iweka described a comparable case of acute subdural hematoma (ASDH) complicating an eclampsia diagnosis [24]. A 19-year-old woman with intrapartum eclampsia at 38 weeks of gestation developed rapidly onset neurological symptoms after delivery. An acute subdural hematoma was diagnosed following a brain CT, and conservative management was opted for due to improvement in the patient’s neurological status. The authors concluded that the favorable outcome of their report demonstrates the importance of a multidisciplinary approach and early detection of ICH, which may complicate eclampsia.

It should be emphasized that there are no universally standardized guidelines specifically for AVM rupture during pregnancy. The management typically aligns with the general principles of handling AVM ruptures and the unique considerations of pregnant patients. Therefore, it is very important to analyze the given clinical cases and further multidisciplinary cooperation in order to develop clear guidelines for the management of such cases.

## 5. Conclusions

Intracranial hemorrhage during pregnancy is a rare and highly dangerous event in which a rapid diagnosis and treatment are key to patients’ survival and long-term well-being. Pregnancy should not be viewed as a barrier to performing angiography or endovascular treatment for vascular causes of ICH. The risks associated with the procedure must be compared with the potential life-saving benefits for the mother. The treatment of a ruptured intracranial aneurysm should be prioritized and conducted urgently, utilizing the best available options regardless of the pregnancy status. This emphasizes the critical nature of treating ruptured aneurysms promptly to optimize maternal outcomes. If the procedure’s timing aligns with a viable gestational age, clinicians should weigh the advantages of performing a concurrent cesarean section to facilitate both maternal and neonatal safety. In cases where the timing corresponds to a pre-viable gestational age, the treatment approach should mirror standard protocols used outside of pregnancy. This is to ensure that maternal safety and outcomes are prioritized above fetal considerations.

The decision-making process regarding the mode of aneurysm treatment and the timing of intervention should involve a multidisciplinary team. This collaborative approach is essential to ensure comprehensive care that considers all aspects of the patient’s health and circumstances.

Currently, there is a lack of follow-up studies investigating the risk of recurrent stroke in subsequent pregnancies after the treatment of a ruptured aneurysm. This underscores the need for further research to enhance clinical decision-making and patient guidance.

Upon diagnosis of a first in a lifetime generalized tonic–clonic seizure in a pregnant patient, there is an extremely high chance of eclampsia etiology of neurological symptoms. Standard treatment should be started immediately following the stabilization of vital signs and rapid transfer to a tertiary center. An interdisciplinary approach to eclampsia treatment is key in maximizing patient survival rates, and establishing a prehospital setting treatment is necessary to enable maternal and fetal stabilization. Ongoing education and training of interdisciplinary medical professionals, including emergency department treatment simulation scenarios, could prepare the team for the unexpected event of treating eclampsia, HELLP syndrome and intracranial hemorrhage.

## Figures and Tables

**Table 1:** Laboratory results upon initial admission to the secondary care emergency department.

Parameter	Value	Unit	Reference Value
Platelets	224	K/μL	140–400
Urinary total protein	3073.4	mg/dL	-
Alanine transaminase	47	IU/L	14–59
Aspartate aminotransferase	55	IU/L	55

**Table 2:** Abnormalities in laboratory tests upon patient admission to the tertiary emergency department.

Parameter	Value	Unit	Reference Value
Platelets	148	×10^9^/L	150–410
Urinary total protein	13.3	g/L	-
Alanine transaminase	122	U/L	<34
Aspartate aminotransferase	252	U/L	11–34
Lactate dehydrogenase	1233	U/L	125–220

**Table 3:** Laboratory test results upon discharge from the tertiary hospital.

Parameter	Value	Unit	Reference Value
Platelets	488	×10^9^/L	150–410
Urinary total protein	0.51	g/L	-
Alanine transaminase	34	U/L	<34
Aspartate aminotransferase	17	U/L	11–34
Lactate dehydrogenase	255	U/L	125–220

## Abbreviations

ICH	Intracranial hemorrhage
WFNS	World Federation of Neurological Surgeons
aSAH	Aneurysmal subarachnoid hemorrhage
OB/GYN	Obstetrics and gynecology
PE	Preeclampsia
HELLP	Hemolysis, elevated liver enzymes and low platelets syndrome
DIC	Disseminated intravascular coagulation
CVST	Cerebral venous sinus thrombosis
AVM	Arteriovenous malformations
BBB	Blood–brain barrier
CBF	Cerebral blood flow
CPP	Cerebral perfusion pressure
MR/MRI	Magnetic resonance imaging
CT	Computer tomography
CNS	Central nervous system
SAH	Subarachnoid hemorrhage
PRES	Posterior reversible encephalopathy syndrome
CS	Cesarean section
